# A Multi-Sensor Tight Fusion Method Designed for Vehicle Navigation

**DOI:** 10.3390/s20092551

**Published:** 2020-04-30

**Authors:** Qifeng Lai, Hong Yuan, Dongyan Wei, Ningbo Wang, Zishen Li, Xinchun Ji

**Affiliations:** 1 Aerospace Information Research Institute, Chinese Academy of Science, Beijing 100864, China; laiqf@aircas.ac.cn (Q.L.); weidy@aircas.ac.cn (D.W.); wangningbo@aoe.ac.cn (N.W.); lizishen@aoe.ac.cn (Z.L.); jixc@aircas.ac.cn (X.J.); 2 School of Electronic, Electrical and Communication Engineering, University of Chinese Academy of Sciences, Beijing 100864, China

**Keywords:** GNSS, INS, odometer, barometric altimeter, clock model, tight fusion

## Abstract

Using the Global Navigation Satellite System (GNSS), it is difficult to provide continuous and reliable position service for vehicle navigation in complex urban environments, due to the natural vulnerability of the GNSS signal. With the rapid development of the sensor technology and the reduction in their costs, the positioning performance of GNSS is expected to be significantly improved by fusing multi-sensors. In order to improve the continuity and reliability of the vehicle navigation system, we proposed a multi-sensor tight fusion (MTF) method by combining the inertial navigation system (INS), odometer, and barometric altimeter with the GNSS technique. Different fusion strategies were presented in the open-sky, insufficient satellite, and satellite outage environments to check the performance improvement of the proposed method. The simulation and real-device tests demonstrate that in the open-sky context, the error of sensors can be estimated correctly. This is useful for sensor noise compensation and position accuracy improvement, when GNSS is unavailable. In the insufficient satellite context (6 min), with the help of the barometric altimeter and a clock model, the accuracy of the method can be close to that in the open-sky context. In the satellite outage context, the error divergence of the MTF is obviously slower than the traditional GNSS/INS tightly coupled integration, as seen by odometer and barometric altimeter assisting.

## 1. Introduction

For vehicle navigation in urban environments, the Global Navigation Satellite System (GNSS) cannot provide continuous and reliable high-precision location services in cases where the transmitted signals are reflected and attenuated by trees and buildings, or even blocked in underground garages and tunnels. Fusing multi-sensors with GNSS is an effective way to improve the availability of the navigation system. The inertial measurement unit (IMU) is the suitable sensor for vehicles, with quickly improved performance and largely reduced cost of inertial sensors, and it is expected that by applying the high-grade IMUs to urban vehicles, the highly accurate clock model can help improve the performance of the GNSS in low satellite visibility conditions. Recently, the GNSS receivers have included a Temperature Compensated Crystal Oscillator (TCXO), characterized by a short-term stability (τ = 1 s) of 1 × 10^−9^ s that leads to a 0.3 m error in the pseudorange. As the chip-scale atomic clock (CSAC) technology is developing rapidly, it can achieve a short-term stability (τ = 1 s) of 2.5 × 10^−12^ s stability, about a 0.075 m error in the pseudorange [1]. The stability is comparable to the traditional Rubidium clock, and the size is just about one hundred milliliters. The atomic chip clock can be employed to improve the GNSS performance. The mileage data of the wheels can be obtained directly from the vehicle, which is helpful to reduce divergence in a long satellite outage environment. The barometric altimeter can provide elevation to distinguish the vehicle on or under the overpass in urban environments. The aforementioned sensors are expected to be widely applied in vehicle navigation in the near future, considering the advantage of easy acquisition for vehicles as well as their low cost.

The research on fusing the aforementioned sensors and GNSS can be mainly divided into two categories. The first is the GNSS/DR (Dead Reckoning sensor), like the GNSS/INS fusion, and the other is GNSS augmented with a barometric altimeter and clock model aiding. In case of the short-term failure of Global Positioning System (GPS) signals, INS can be used to determine position and orientation by integrating acceleration and angular velocity. Previous research has been carried out on three GNSS/INS integration types: the loosely coupled, tightly coupled, and ultra-tight integration [2,3,4]. The ultra-tight integration is suitable for highly dynamic cases or weak signal environments, but the mobility of vehicles is relatively low. In addition, the integration needs to adjust the receiver tracking loop, and it is not accessible when using commercial receivers. The main difference between loosely and tightly coupled integration is the measurement type. For loosely coupled integration, the measurements are the position and velocity, while they are pseudoranges and doppler shifts for the tightly coupled integration. The tightly coupled integration is more suitable in case of reduced satellite signal availability [5,6,7,8]. Recently, as raw observations retrieved from the GNSS chipsets in smart devices are now available to the users and developers, a number of studies on low-cost, tightly coupled GNSS/INS have also been carried out, and they were found to have good performance [9,10]. However, the position error of INS accumulates rapidly during the period of GNSS outage, so that the GNSS/INS fusion cannot provide position service in the indoor environment for a long time.

The odometer is added to the GNSS/INS system to solve the problem of the quadratic growing for position error, when there is a long satellite outage. The velocity of vehicles can be obtained by the odometer, and the position is calculated by one integration of velocity [11]. The divergence of the odometer is slower than INS. The GPS/IMU/odometer integration systems were designed using different filters to improve the position performance of the system. Falco et al. designed a GPS/IMU/odometer tightly coupled system through the use of a Kalman Filter (KF) [12]. Liu also used the KF to integrate GPS, BDS (BeiDou Navigation Satellite System), MEMS-INS (Micro-Electro-Mechanical System-Inertial Navigation System), and odometer sensors for real-time high-precision vehicle positioning in an urban degraded and denied environment [13]. Georgy designed a GPS/IMU/odometer integration system using a mixture particle filter [14]. Zengke designed a GPS/INS/odometer system based on a Fuzzy Neural Network (FNN), using odometer velocity correction to enhance resolution accuracy over a long GPS outage [15]. The test results report that the odometer can help to increase the positioning performance effectively, and the KF is more suitable for practical applications due to its low complexity and good performance. However, there are two disadvantages for the GPS/INS/odometer system: one is the quick height divergence when there is a satellite outage, and the other is that the fusion algorithms only consider two cases with and without satellites, and the insufficient satellites case is not specially treated and improved. 

The barometric altimeter can help to solve the above problems. The local height can be calculated by the relationship between local pressure and reference pressure, which is generally standard atmosphere. The standard atmosphere is the mean sea-level atmospheric pressure, where the height is zero. The standard atmosphere is different in various regions, and changes over time. Parviainen et al. calculates the user’s altitude by a differential way in personal navigation [16]. This method needs to set up a reference station, and its baseline is not very long. Yen treats elevation as a geocentric satellite and combines it with available GPS to estimate position in the GNSS signal-degraded environment [17]. The research of combining barometric with GPS/INS/odometer is rather limited. Chiang et al. simply treats the barometer height as a measurement in the KF in the INS/GNSS/odometer/barometer integration system [18], without using advantages of the barometer treated as a geocentric satellite in tightly coupled integration. Moreover, in this paper, the pressure at the initial position is treated as the reference pressure, and it is not a proper method, for the pressure tendency changes a lot when the vehicle is far from the initial position and also varies with time.

The quality of GNSS receiver clocks also exhibit high impacts on the performance of positioning in the complex environment [19,20]. Krawinkel assessed the benefits of receiver clock modeling in code-based GNSS navigation, and achieved position solutions with only three satellites in view [21]. Li employed chip-scale atomic clock (CSAC) to assist BDS positioning, and the clock prediction accuracy achieved 3 ns in 60 min [22]. These results show the improvement in GNSS position brought by a high-stability clock and analyze the influence of satellite geometry range on clock model-assisted positioning. However, the recent research mainly focuses on the clock model-assisted method in GNSS position, and few works discuss the clock model affection in the GNSS/INS integration system.

In urban navigation, the vehicle always travels along open-sky, insufficient satellites, and satellite outage environments [23]. Paul D points out that in challenging environments, the context is critical to the operation of a navigation or positioning system [24]. Saeedi uses low-cost sensors to improve the accuracy and robustness of a context-aware navigation system for pedestrian positioning, considering the person activity and placement context [25]. However, few public literatures focus on the context-based vehicle fusion position. 

The purpose of this paper is to report a method systematically fusing the GNSS, INS, odometer, barometric, and clock model, named the multi-sensor tight fusion (MTF) method, for vehicle navigation in urban environments. It designs the different strategies for vehicles traveling along open-sky, insufficient satellites, and satellite outage contexts. In addition, to reduce the cost of setting up a reference station, the method compensates the error of the barometric altimeter when there are good satellite visibility conditions. Simulation and real-device test results show that the MTF method is significantly better than the traditional GNSS/INS tightly coupled integration in challenging urban environments. Following this introduction, the MTF method is presented along with the description of the observation model of all included sensors (GNSS, INS, odometer, barometric, and clock). The reference pressure is estimated by Kalman Filter (KF), to calculate the barometric height. The performance of the proposed MTF method is then validated in both simulated and real-device experiments under different environments. Finally, the summary and conclusions are given.

## 2. Methods

### 2.1. Observation Model of Sensors

The observation model of the GNSS, odometer, and barometric altimeter are described as follows. The GNSS pseudorange measurement, $\rho_{i}$, between satellite and receiver can be expressed as [26]

(1) $$\rho_{i}=\sqrt{{\left(x-x^{i}\right)}^{2}+{\left(y-y^{i}\right)}^{2}+{\left(z-z^{i}\right)}^{2}}+t_{u}$$

where x,y, and z are three-dimensional coordinates of the user position in the Earth-centered Earth-fixed (ECEF) coordinate system, and $t_{u}$ is the receiver clock offset. $x^{i}$,$y^{i}$, and $z^{i}$ are three-dimensional coordinates of the *i*-th satellite position in the ECEF coordinate system, and four or more satellites are needed to estimate the user’s position. The pseudorange rate, ${\dot{\rho }}_{i}$, is given as follows [26].

(2) $${\dot{\rho }}_{i}=e_{ix}\left(\dot{x}-{\dot{x}}^{i}\right)+e_{iy}\left(\dot{y}-{\dot{y}}^{i}\right)+e_{iz}\left(\dot{z}-{\dot{z}}^{i}\right)+dt_{u}$$

with the notation $\dot{x}$, $\dot{y}$, and $\dot{z}$ for three-dimensional coordinates of the user velocity in the ECEF coordinate system, $dt_{u}$ for clock drift, and ${\dot{x}}^{i}$,${\dot{y}}^{i}$, and ${\dot{z}}^{i}$ for three-dimensional coordinates of the i-th satellite velocity in the ECEF coordinate system. The line-of-sight vector, $e_{i}$, is expressed as $e_{ix}=\left(x-x^{i}\right)/r^{i}$, $e_{iy}=\left(y-y^{i}\right)/r^{i}$, $e_{iz}=\left(z-z^{i}\right)/r^{i}$, and $r^{i}=\sqrt{{\left(x-x^{i}\right)}^{2}+{\left(y-y^{i}\right)}^{2}+{\left(z-z^{i}\right)}^{2}}$. The observations of INS are position, velocity, and attitude and the calculation from IMU measurement, acceleration, and angular velocity can be viewed in Reference [27].

The odometer measurement is the vehicle velocity, $v_{ODO}^{}$, in the body frame, and the observation equation is [14,15]

(3) $$v_{ODO}^{}=sf_{{}_{ODO}}\cdot C_{n}^{b}\cdot v^{n}{}_{3\times 1}$$

where the forward velocity of $v_{ODO}^{}$ is measured by the odometer, and the lateral and up velocity of $v_{ODO}^{}$ are supposed as zero, considering the Non-Holonomic Constraint (NHC). $v^{n}{}_{3\times 1}$ is the velocity of user in the local frame, $C_{n}^{b}$ is the rotation matrix from the local frame to the body frame, and $sf_{{}_{ODO}}$ is the scale factor.

The measurement of the barometric altimeter is local pressure, P, and differential Equation (4) describing the altitude, H, as a function of the local pressure, P, using the isothermal atmospheric model of the US Standard Atmosphere [28,29].

(4) $$H-H_{0}=\frac{RT}{g}\ln\frac{P_{0}}{P}=18,410*\left(1+\frac{t}{273.15}\right)*\mathrm{lg}\frac{P_{0}}{P}$$

when $H_{0}$ is equal to zero, $P_{0}$ is the standard atmospheric pressure. The standard atmospheric pressure varies in different regions and changes continuously over time. In Equation (4), R is the specific gas constant equal to $R^{*}/m$, $R^{*}$ is the ideal gas constant equal to 8.31446 J/K/m°l, m is the average molecular weight equal to 28.9634 g/m°l, g is the standard gravity value equal to 9.80665 $m/s^{2}$, and t is the local temperature in the unit of Celsius. 

In case the barometric altimeter is taken as a geocentric satellite, the observation equation can be written as [26]:

(5) $$\frac{x^{2}+y^{2}}{{\left(R_{e}+h_{BARO}\right)}^{2}}+\frac{z^{2}}{{\left(R_{p}+h_{BARO}\right)}^{2}}=1$$

where $h_{BARO}$ is the barometric measurement, and is calculated by Equation (4). The clock model measurement is the predicted clock error, $t_{prediction}$, and the observation equation is

(6) $$t_{prediction}=t_{u}$$

with the notation $R_{e}$ for the semi-major axis of earth, and $R_{p}$ for the semi-minor axis.

### 2.2. Fusion Method Design

**Figure 1:**
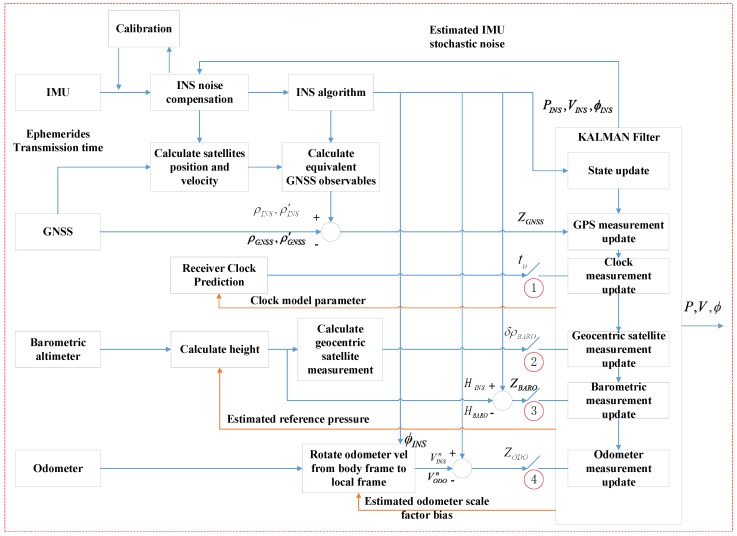
Flow chart of the multi-sensor tight fusion (MTF) method.

The proposed MTF method is illustrated in Figure 1. It uses a sequential Extended Kalman Filter (EKF), and the EKF is widely applied in practical projects [12]. The MTF method considers the observation model mentioned in Section 2.1 to design the KF. In the KF, the measurements of each sensor update sequentially. Based on traditional GNSS/INS tightly coupled integration, the MTF method adds the odometer and barometric altimeter sensor to the filter. The MTF method designs different strategies for distinct environments, such as the open-sky, insufficient satellite, satellite outage cases. In the open-sky context, the KF carries out altimeter and odometer measurement updates by turning on switches 3 and 4, indicated by the red circle in Figure 1. As the accuracy of GNSS is high, the errors of sensors can be estimated correctly, which are used to compensate the measurement and improve position performance when the GNSS signal is impaired. In the insufficient satellite context, the KF turns on switches 1 and 2, and turns off switches 3 and 4. The barometric altimeter, treated as a geocentric satellite, is used for measurement updates with the remaining satellites and clock model. The clock model is used to predict the clock error. In the satellite outage case, the KF turns on switches 3 and 4, and turns off switches 1 and 2. The odometer and barometric altimeter are used to reduce the horizontal and height position error divergence, respectively.

In Part I of Figure 1, for the IMU, the block labeled as INS noise compensation is used to compensate the bias of the accelerometer and gyro. The INS algorithm processes the inertial navigation solution, which can calculate position, velocity, and altitude.

For the GNSS, the block labeled as calculate equivalent GNSS observables is used to calculate the equivalent pseudorange and pseudorange rate using the position, velocity of the satellite, and INS. The pseudorange and the pseudorange rate difference between GNSS and INS is the KF measurement. 

For the odometer, the scale factor is used to compensate the forward velocity measurement. The velocity difference between the odometer and INS in the local frame is the KF measurement.

For the barometric altimeter, calculate height calculates the local height by the relationship between local pressure and reference pressure, which is generally standard atmosphere. The standard atmosphere is the mean sea-level atmospheric pressure, and where the height is zero. In the MTF method, the reference pressure is estimated by the KF. The height difference between barometric height and INS is the KF measurement. In addition, the barometric height also can be used to calculate the geocentric satellite measurement, which is used in the insufficient satellite context.

### 2.3. Fusion Filter Design

In the KF, the dynamic state-space linear model is expressed as [12,27]

(7) $$\delta \dot{x}=\phi \cdot \delta x+\upsilon$$

(8) Z=H⋅δx+e

where notation δx is for the state, υ is for the process noise, ϕ is for the system transition matrix, Z is for the KF measurement, H is for the measurement matrix, and e is for the measurement noise. The filter state δx includes the system state and the error of sensors. The system states are altitude, velocity, and position.

(9) $$\delta x=\left[\begin{array}{ccccc} \begin{array}{ccccc} \delta \phi_{3\times 1}; & \delta v_{3\times 1}; & \delta p_{3\times 1}; & \delta \omega_{3\times 1}; & \delta f_{3\times 1}; \end{array} & t_{u}; & dt_{u}; & \delta sf_{odo}^{}; & lb \end{array}\right]$$

where, $\delta \phi_{3\times 1}$ is the altitude error vector in the local frame, $\delta v_{3\times 1}$ is the velocity error vector in the local frame, $\delta p_{3\times 1}$ is the position error vector in the local frame, $\delta \omega_{3\times 1}$ is the error vector related to the angular rates measured by the IMU in the body frame, $\delta f_{3\times 1}$ is the error vector related to the specific forces measured by the IMU in the body frame, $t_{u}$ is the receiver’s clock bias, where the unit is meters, $dt_{u}$ is the receiver’s clock drift, where the unit is meters/second, $\delta sf_{odo}^{}$ is the error related to the odometer scale factor, and lb is the estimated reference atmospheric pressure.

(10) $$\phi =\left[\begin{array}{ccccccccc} M_{aa} & M_{av} & M_{ap} & -C_{b}^{n} & 0_{3\times 3} & 0_{3\times 1} & 0_{3\times 1} & 0_{3\times 1} & 0_{3\times 1}\\ M_{va} & M_{vv} & M_{vp} & 0_{3\times 3} & C_{b}^{n} & 0_{3\times 1} & 0_{3\times 1} & 0_{3\times 1} & 0_{3\times 1}\\ 0_{3\times 3} & M_{pv} & M_{pp} & 0_{3\times 3} & 0_{3\times 3} & 0_{3\times 1} & 0_{3\times 1} & 0_{3\times 1} & 0_{3\times 1}\\ 0_{3\times 3} & 0_{3\times 3} & 0_{3\times 3} & -diag(1/\tau_{g1\times 3}) & 0_{3\times 3} & 0_{3\times 1} & 0_{3\times 1} & 0_{3\times 1} & 0_{3\times 1}\\ 0_{3\times 3} & 0_{3\times 3} & 0_{3\times 3} & 0_{3\times 3} & -diag(1/\tau_{a1\times 3}) & 0_{3\times 1} & 0_{3\times 1} & 0_{3\times 1} & 0_{3\times 1}\\ 0_{1\times 3} & 0_{1\times 3} & 0_{1\times 3} & 0_{1\times 3} & 0_{1\times 3} & 0 & 1 & 0 & 0\\ 0_{1\times 3} & 0_{1\times 3} & 0_{1\times 3} & 0_{1\times 3} & 0_{1\times 3} & 0 & 0 & 0 & 0\\ 0_{1\times 3} & 0_{1\times 3} & 0_{1\times 3} & 0_{1\times 3} & 0_{1\times 3} & 0 & 0 & 0 & 0\\ 0_{1\times 3} & 0_{1\times 3} & 0_{1\times 3} & 0_{1\times 3} & 0_{1\times 3} & 0 & 0 & 0 & 0 \end{array}\right]$$

where notation $\tau_{a1\times 3}$ and $\tau_{g1\times 3}$ represent the time constant vectors of the Gauss–Markov noises related to the accelerometers and gyroscopes, respectively. The M matrixes are shown in the equations in the Appendix A.

The GNSS observation model is linearized through the first-order Taylor expansion, then the measurement vector and the measurement matrix are expressed as

(11) $$Z_{GNSS}=\left[\begin{array}{c} \rho_{INS}^{1}-\rho_{GNSS}^{1}\\ \rho_{INS}^{2}-\rho_{GNSS}^{2}\\ \ldots \\ \rho_{INS}^{n}-\rho_{GNSS}^{n} \end{array}\right]$$

(12) $$H_{GNSS}=\left[\begin{array}{cccc} \frac{x-x^{1}}{r_{1}} & \frac{y-y^{1}}{r_{1}} & \frac{z-z^{1}}{r_{1}} & 1\\ \frac{x-x^{2}}{r_{2}} & \frac{y-y^{2}}{r_{2}} & \frac{z-z^{2}}{r_{2}} & 1\\ \ldots & \ldots & \ldots & 1\\ \frac{x-x^{n}}{r_{n}} & \frac{y-y^{n}}{r_{n}} & \frac{z-z^{n}}{r_{n}} & 1 \end{array}\right]$$

using the notation n for the number of satellites. The odometer observation model is linearized through the first-order Taylor expansion, then the measurement vector and the measurement matrix can be written as

(13) $$Z_{ODO}^{}=sf_{{}_{ODO}}\cdot v_{INS}^{n}-C_{b}^{n}v_{ODO}^{}$$

(14) $$H_{ODO}=\left[\begin{array}{cc} \begin{array}{cc} \begin{array}{cc} \begin{array}{ccccc} -v_{ODO}\times & diag{\left(sf_{{}_{ODO}}\right)}_{3\times 3} & 0_{3\times 3} & 0_{3\times 3} & 0_{3\times 3} \end{array} & 0_{3\times 2} \end{array} & {\left(v_{INS}^{n}\right)}_{3\times 1} \end{array} & 0_{3\times 1} \end{array}\right]$$

The barometric altimeter observation model is linearized through the first-order Taylor expansion, then the measurement and the measurement matrix are expressed as:

(15) $$Z_{BARO}=H_{INS}-\left(H_{0}+18,400*\left(1+\frac{t}{273.15}\right)*\mathrm{lg}\frac{P_{0}}{P}\right)$$

(16) $$H_{BARO}=\left[\begin{array}{cc} \begin{array}{cc} \begin{array}{cc} \begin{array}{ccccc} 0_{1\times 3} & 0_{1\times 8} & 1 & 0_{1\times 3} & 0_{1\times 3} \end{array} & 0_{1\times 2} \end{array} & 0 \end{array} & 18,400*\left(1+\frac{t}{273.15}\right)*\frac{1}{P_{0}\ln(10)} \end{array}\right]$$

The GNSS/barometric altimeter/clock observation models are linearized through the first-order Taylor expansion, then the measurement and the measurement matrix are expressed as

(17) $$Z_{sat2}={\left[\begin{array}{cccc} \delta \rho^{1} & \delta \rho^{2} & \delta \rho_{BARO} & t_{prediction} \end{array}\right]}^{T}$$

(18) $$\delta \rho_{BARO}=\frac{1}{2}\left(\frac{x_{INS}^{2}+y_{INS}^{2}}{{\left(R_{e}+h_{BARO}\right)}^{2}}+\frac{z_{INS}^{2}}{{\left(R_{p}+h_{BARO}\right)}^{2}}-1\right)$$

(19) $$H_{sat2}=\left[\begin{array}{cc} \begin{array}{cc} \begin{array}{cc} \begin{array}{ccccc} 0_{4\times 3} & 0_{4\times 3} & M_{1}*C_{n}^{e} & 0_{4\times 3} & 0_{4\times 3} \end{array} & M_{2} \end{array} & 0_{4\times 1} \end{array} & 0_{4\times 2} \end{array}\right]$$

(20) $$M_{1}=\left[\begin{array}{ccc} \frac{x-x^{1}}{r_{1}} & \frac{y-y^{1}}{r_{1}} & \frac{z-z^{1}}{r_{1}}\\ \frac{x-x^{2}}{r_{2}} & \frac{y-y^{2}}{r_{2}} & \frac{z-z^{2}}{r_{2}}\\ \frac{x}{{\left(R_{e}+h_{BARO}\right)}^{2}} & \frac{y}{{\left(R_{e}+h_{BARO}\right)}^{2}} & \frac{z}{{\left(R_{p}+h_{BARO}\right)}^{2}}\\ 0 & 0 & 0 \end{array}\right]$$

(21) $$M_{2}={\left[\begin{array}{cccc} 1 & 1 & 0 & 1 \end{array}\right]}^{T}$$

where $\delta \rho^{1}$ is the GNSS measurement, the same as in Equation (11), $t_{prediction}$ is the predicted clock error, and $C_{n}^{e}$ is the rotation matrix from the local frame to the ECEF frame.

## 3. Simulation Test

To validate the proposed MTF method, a vehicle traveling scenario is simulated, and the associated sensor measurements, i.e., IMU, GNSS, odometer, and barometric altimeter, are generated accordingly. As the sensor errors are known in advance in the simulation, they can be used to evaluate the estimated sensor errors (including accelerometer bias, gyroscope bias, odometer scale factor, reference pressure of barometric altimeter, and GNSS clock model) in the KF in the open-sky case. Only if the estimated sensor errors converge to the right value soon will the position in insufficient satellite and satellite outage cases perform well. 

**Figure 2:**
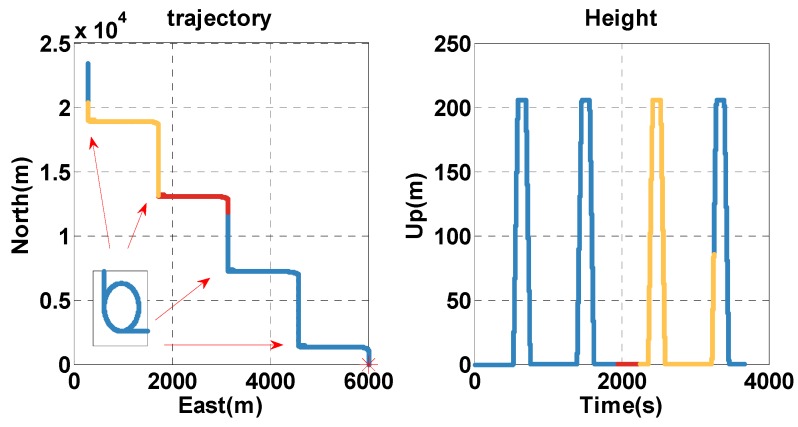
Horizontal trajectory and height of the simulation test. The blue, red, and yellow lines indicate the open-sky, insufficient satellite, and satellite outage context, respectively.

The actions of vehicles include stationary, uniform speed, acceleration, deceleration, turning, climbing, and downhill. The total simulation period covers 3664 s. The simulated trajectory is shown in Figure 2, where the red star is the start point. The initial position, velocity, and altitude are given in Table 1. GNSS measurements are pseudoranges and pseudorange rates, which are computed by combing the position and velocity of the vehicle with that of satellites, obtained from GPS ephemeris on 21 November 2019. The Gaussian noise of 1 m is added to the pseudorange, and the Gaussian noise of 0.1 m/s is added to the pseudorange rate. The clock model is also added to the pseudorange and pseudorange rate. The clock drift is constant at 1 × 10^−8^, which is equivalent to 2.99792458 m/s, and the Gaussian noise of 1 × 10^−11^ is added to the clock drift. The initial clock error is zero. The IMU measurements are the specific force and angular rate from the accelerometer and gyroscope, respectively. The bias of 100 ug and velocity random walk of 10 $ug/\sqrt{Hz}$ is added to the specific force. The bias of 0.05 ${}^{{}^{\circ}}/hr$ and velocity random walk of 1 × 10^−3^ ${}^{{}^{\circ}}/\sqrt{hr}$ is added to the angular rate. The odometer measurement is forward velocity, and its scale factor is 0.9. The Gaussian noise of 0.1 m/s is added to the velocity. The barometric altimeter measurement is local pressure, and reference pressure is 1000 hPa, where the height is zero. The Gaussian noise of 0.1 hPa is added to local pressure. Table 2 lists the simulation parameters of each sensor.

**Figure 3:**
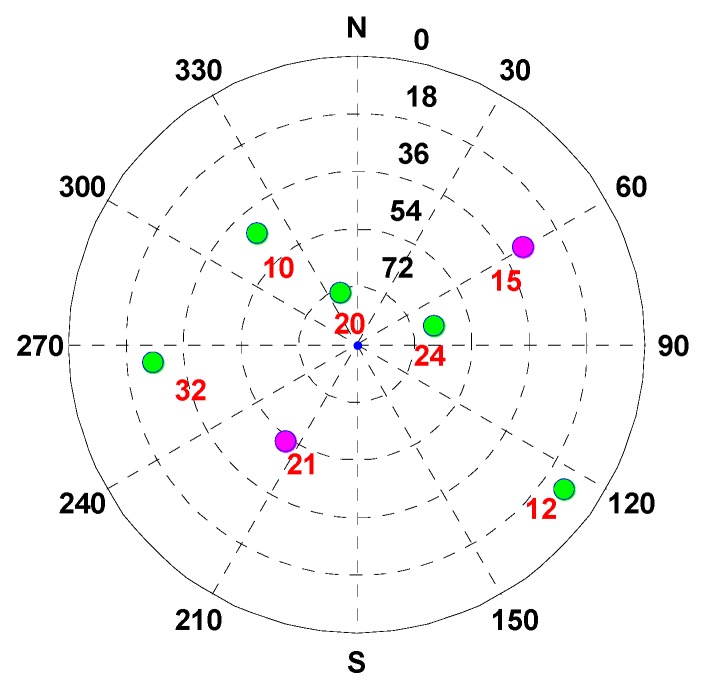
The sky plot of satellites for the epoch 1950 s, in which the green and magenta dots denote the blocked and visible satellites, respectively.

In the simulation test, the vehicle is driven along the contexts, such as open-sky, insufficient satellite, and satellite outage. To analyze the performance of the MTF method in the above contexts, the test time is divided into: during 0–1949 and 3251–3664 s, the vehicle is in the open-sky area, where the GNSS exhibits a high accuracy. The experiment analysis focuses on the error states of each sensor estimated by the filter, and that is significant for position resolution when the GNSS signal is blocked. During 1950–2249 s, the vehicle is in the area of the insufficient satellite, where the number of visible satellites decrease to 2. In Figure 3, the magenta dots indicate the remaining satellites at 1950 s. The MTF method achieves measure-up by combining the remaining satellites with the barometer altimeter and the clock model. The analysis is conducted in terms of position and velocity errors comparing the proposed MTF method and the traditional GNSS/INS tightly coupled integration. During 2250–3250 s, the vehicle is in the satellite outage area. The MTF method carries on dead reckoning based on INS, odometer, and barometric altimeter. The performance of the MTF method is also compared with traditional GNSS/INS tightly coupled integration.

The noise parameters used in the filter are set as follows. The process noise is set as 1 × 10^−3^ ${}^{{}^{\circ}}/\sqrt{\mathrm{hr}}$ for the altitude error state and 10 $\mathrm{ug}/\sqrt{\mathrm{Hz}}$ for the velocity error state. The measurement noises are 1 m and 0.1 m/s for GNSS pseudorange and pseudorange rate, 0.1 m/s for odometer velocity, 1 m for barometric height, and 2.99 × 10^−3^ m for the clock model, respectively.

### 3.1. Open-Sky Case 

**Figure 4:**
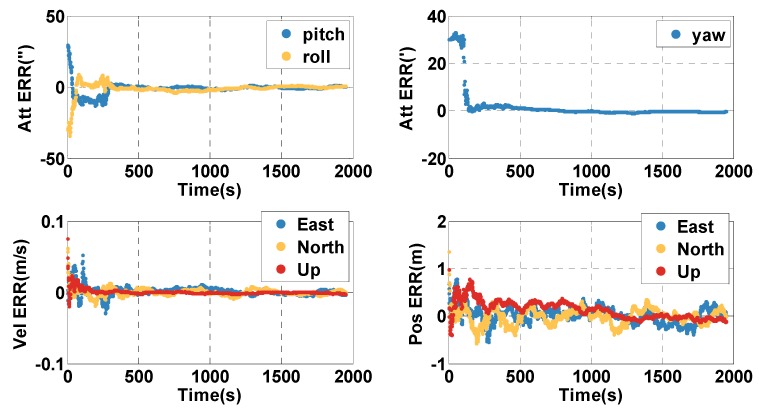
The position (Pos), velocity (Vel), and attitude (Att) errors (ERR) in the open-sky case by applying the MTF method.

In the open-sky context (0–1949 s), the position and velocity errors of the MTF method are shown in Figure 4. The position and velocity errors are presented in the east, north, and up directions, and in the first 100 s, the vehicle is stationary. The position and velocity errors converge at the beginning. However, the attitude errors decrease quickly when the vehicle starts to move. To statistically analyze the performance of the proposed MTF method, the errors are characterized by the 67th percentile. The orientation error is 0.46 min, and the pitch and roll errors are 0.94 s and 0.90 s. The horizontal position error is 0.22 m, and the height error is 0.20 m. The horizontal velocity error is 0.005 m/s, and the vertical velocity error is 0.0015 m/s. The performance of the fusion system is much better than GNSS alone by filtering out the noise on the pseudorange and the pseudorange rate. 

**Figure 5:**
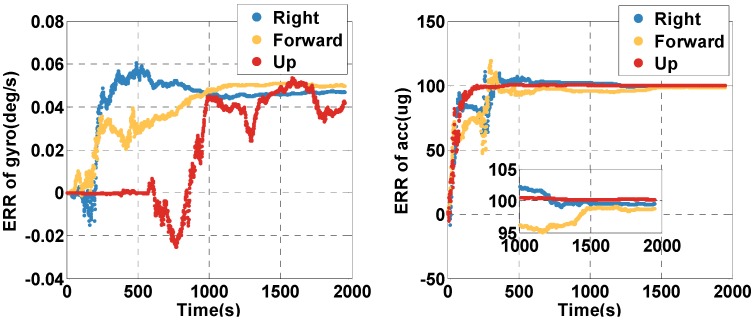
The estimated angular rate and specific force bias of gyro and accelerometer (acc) in the open-sky case. ERR = Error.

Supposing the INS noise compensation module in Figure 1 stops working, the estimated biases of the gyro and accelerometer are shown in Figure 5. For the gyro, the errors in the right and forward direction, which is related to horizontal altitude, converge to the final value (0.05 degree/s shown in Table 2) in about 250 s. The convergence time of error in the up direction is much longer, about 1000 s, which is related to orientation. For the accelerometer, the error in the up direction, which is related to height, converges to the final value (100 ug, shown in Table 2) in about 100 s. The convergence time of errors in the right and forward direction is much longer, about 260 s, which is related to horizontal movement.

**Figure 6:**
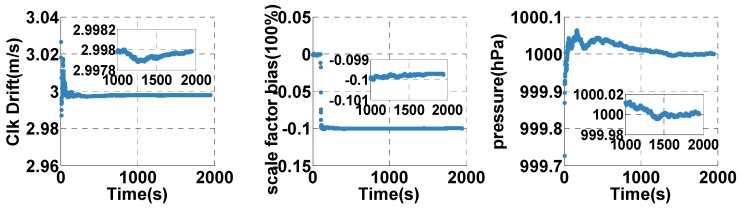
The estimated clock (Clk) drift, error of the odometer’ scale factor, and reference pressure in the open-sky case.

The estimated clock drift, error of the odometer’s scale factor, and reference pressure are shown in Figure 6. The estimated clock drift quickly converges to the final value (2.9979 m/s, shown in Table 2). The estimated scale factor error of the odometer converges to the final value (−0.1), consistent with the scale factor in Table 2. The reference pressure converges to the final value (1000 hpa, shown in Table 2). Therefore, the sensor errors can be estimated correctly in the open-sky context.

### 3.2. Insufficient Satellite Case

**Figure 7:**
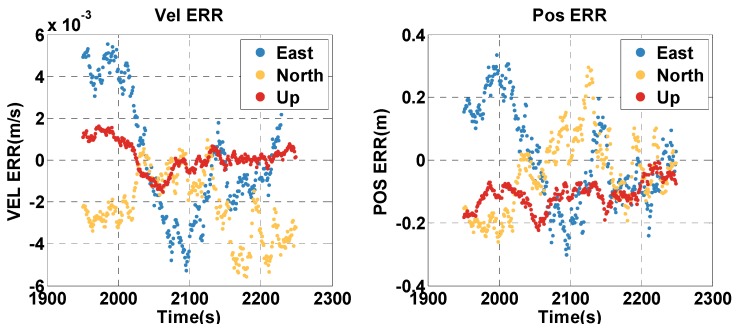
The position and velocity errors of the MTF method in the insufficient satellite case.

**Figure 8:**
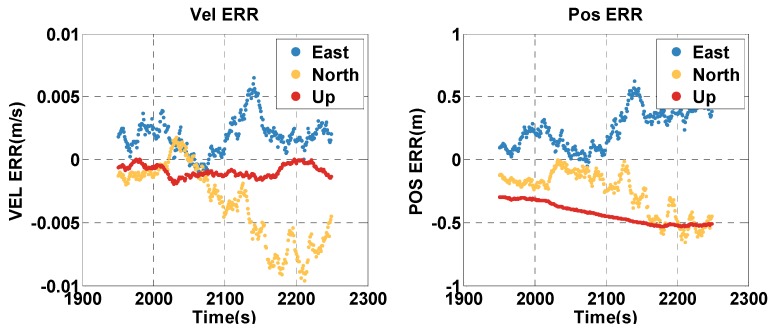
The position and velocity errors of the traditional GNSS/INS tightly coupled integration solution in the insufficient satellite case.

The vehicle travel in the insufficient satellite context lasts for 6 min (1950–2249 s). As shown in Figure 3, the remaining visible satellites are presented in red circles for the epoch of 1950 s. The position and velocity errors of the MTF method in the east, north, and up directions are shown in Figure 7. The errors of the traditional GNSS/INS tightly coupled integration are shown in Figure 8. The errors of the proposed method do not grow over 6 min in Figure 7, but the errors of GNSS/INS tightly coupled integration increase in Figure 8, especially the position error. In the same time, Figure 7 shows that the error of the MTF method is unstable at the beginning of the period, and the reason is that the new measurements update causes slight fluctuation in the filter. The error statistics are shown in Table 3, and they are characterized by the 95th percentile. Comparing the MTF method with GNSS/INS, the horizontal position errors of the two methods are approximately 0.3417 m against 0.6999 m, the height errors are approximately 0.1851 m against 0.5227 m, the horizontal velocity errors are approximately 0.0056 m/s against 0.0087 m/s, and the vertical velocity errors are approximately 0.0014 m/s against 0.0015 m/s. The accuracy of the MTF method is close to that in the open-sky context, and performs better than GPS/INS tightly coupled integration.

### 3.3. Satellite Outage Case

**Figure 9:**
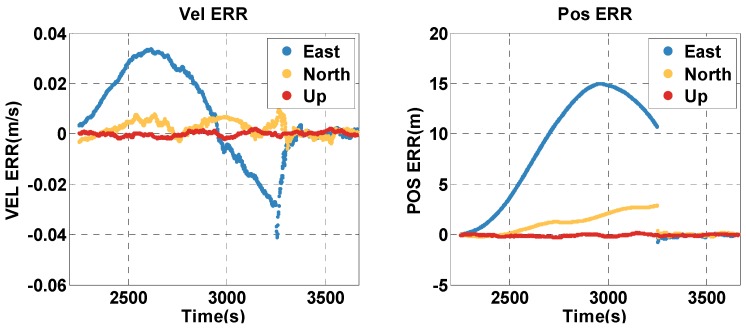
The position and velocity errors of the proposed MTF method in the satellite outage context.

**Figure 10:**
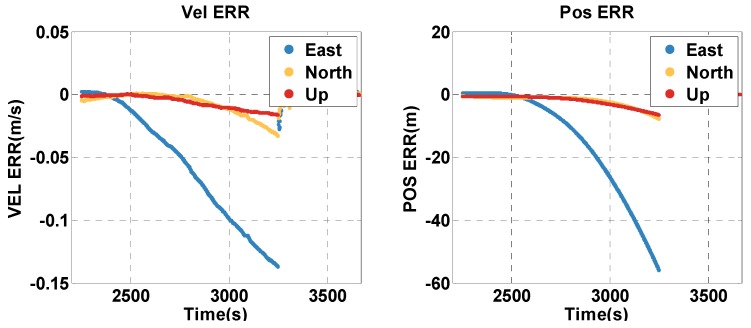
The position and velocity errors of the traditional GNSS/INS tightly coupled integration in the satellite outage case.

The vehicle travels in the satellite outage context for about 16 mins (2250–3250 s). The position and velocity errors of the MTF method are shown in Figure 9. The errors of the traditional GNSS/INS tightly coupled integration are shown in Figure 10. The errors of both methods increase over time, but that of the MTF method is much slower. In addition, the MTF height error does not grow over time. The error statistics are shown in Table 4. The errors are characterized by the 95th percentile. Comparing the MTF method with GNSS/INS, the horizontal position errors of the two methods are approximately 15.04 m against 49.76 m, the height errors are approximately 0.214 m against 5.713 m, the horizontal velocity errors are approximately 0.0332 m/s against 0.1337 m/s, and the vertical velocity errors are approximately 0.0017 m/s against 0.0147 m/s. The MTF method has obvious advantages in both the horizontal and height direction by the aiding of the odometer and barometric altimeter.

## 4. Real-Device Test

**Figure 11:**
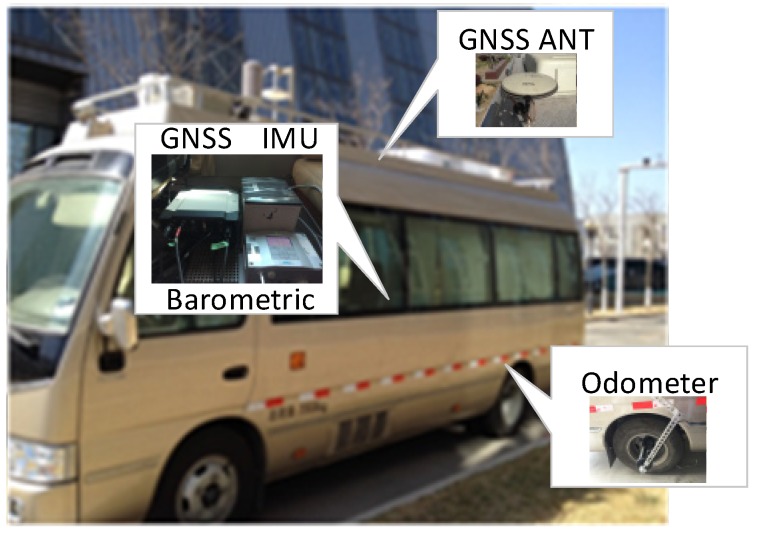
The test platform including GNSS receiver, IMU, barometric altimeter, and odometer.

In the experiment test, the sensor measurements are obtained by a test platform in Figure 11, that consists of the GNSS receiver (Novatel ProPak6 receiver and Novatel GPS-704 antenna), IMU (gyro bias stability: 0.5 ${}^{{}^{\circ}}/\sqrt{\mathrm{hr}}$, angular random walk: 0.012 ${}^{{}^{\circ}}/\sqrt{\mathrm{hr}}$; accelerometer bias stability: 1250 ug, velocity random walk: 100 $\mathrm{ug}/\sqrt{\mathrm{Hz}}$), barometric altimeter (VAISALA PTU 301, resolution: 0.01 hPa), and an odometer (1000 pulse per wheel round). The antenna of the GNSS is mounted on board a vehicle, and the odometer is on the right rear wheel. The sensor measurements are collected on the laptop, and Real-Time Kinematic (RTK) + Strap-down Inertial Navigation System (SINS) results are processed by Inertial Explorer (IE) Post-Processing software (Novatel) are used as a highly precise reference to evaluate the MTF method.

**Figure 12:**
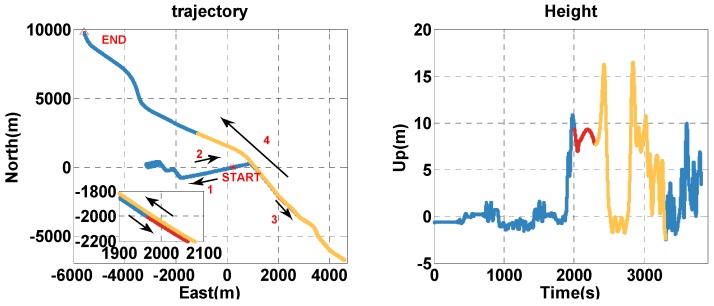
The horizontal trajectory and height of the real-device test. The blue, red, and yellow lines indicate the open-sky, insufficient satellite, and satellite outage parts, respectively.

**Figure 13:**
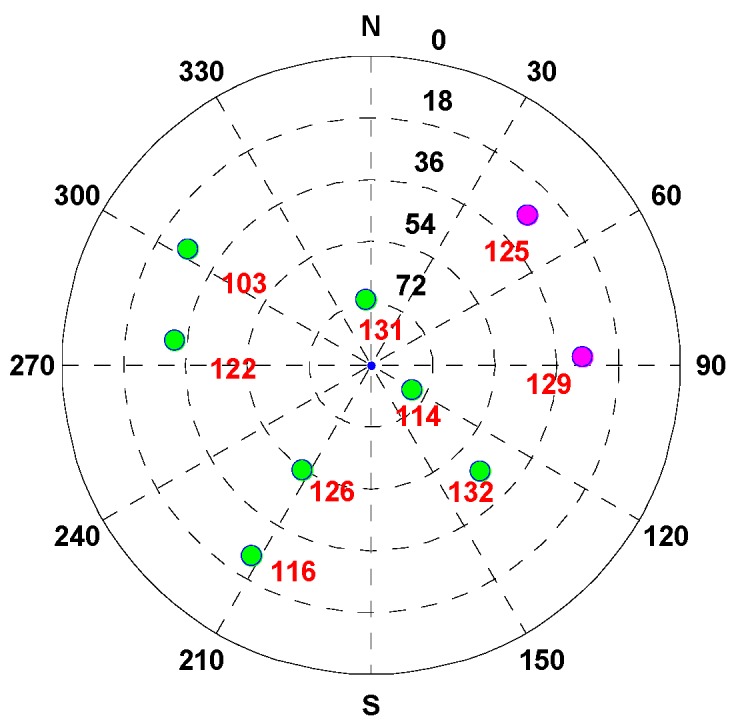
The sky plot of satellites for the epoch of 2000 s, in which green and magenta dots denote the blocked and visible satellites, respectively.

The test lasts for 3803 s. The trajectory is shown in Figure 12, and the vehicle is driven along the 1-2-3-4 arrow indication. To analyze the performance of the MTF method in different contexts, the test time is divided into: during 0–1999 s and 3300–3803 s, the vehicle is in the open-sky context, and the visible satellites number is almost more than 4. During 2000–2299 s, the vehicle is in the insufficient satellite context, and two satellites are left, simulating when the GNSS signal is obscured by the building on one side of the road. In Figure 13, the red circles indicate the remaining satellites at 2000 s. During 2300–3299 s, the vehicle is in the satellite outage context, and there is no visible satellite simulating the tunnel. The analysis is conducted on error comparison between the MTF method and traditional GNSS/INS tight-coupled integration.

The noise parameters used in the filter are set as follows. The process noise is set as 0.0012 ${}^{{}^{\circ}}/\sqrt{\mathrm{hr}}$, for the altitude error state and 1250 $\mathrm{ug}/\sqrt{\mathrm{Hz}}$ for the velocity error state. The measurement noises are sin(EL)/10 m and sin(EL)/100 m/s for GNSS pseudorange and pseudorange rate, 0.1 m/s for odometer velocity, 1 m for barometric height, and 1 × 10^−3^ m for the clock model, respectively.

### 4.1. Open-Sky Case 

**Figure 14:**
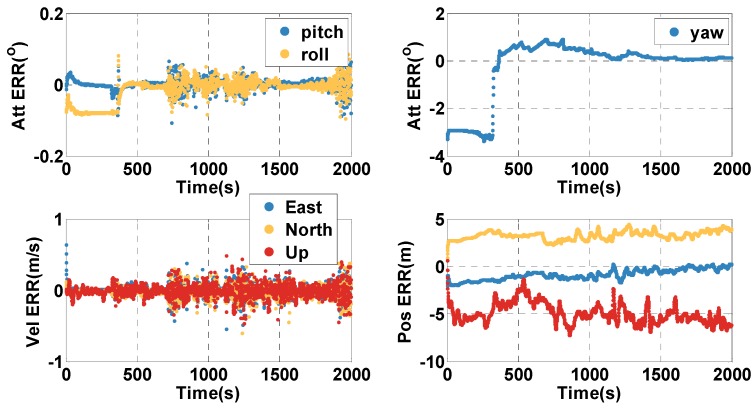
The position, velocity, and attitude errors of the MTF method in the open-sky case.

In the open-sky context (0–1999 s), the position and velocity errors of the MTF method are shown in Figure 14. The position and velocity errors are in the east, north, and up directions, and in the first 300 s, the vehicle is stationary. The position and velocity errors converge at the beginning, and the altitude errors converge after the vehicle moves. To statistically analyze the errors of the MTF method characterized by the 67th percentile, the orientation error is 0.55°, and the pitch and roll errors are 0.009° and 0.017°. The horizontal position error is 3.6 m and the height error is 5.5 m. The horizontal velocity error is 0.08 m/s and the vertical velocity error is 0.07 m/s. The performance of the fusion system is much better than GNSS alone by filtering out the Gaussian noise on the pseudorange and pseudorange rate.

### 4.2. The Insufficient Satellite Case

**Figure 15:**
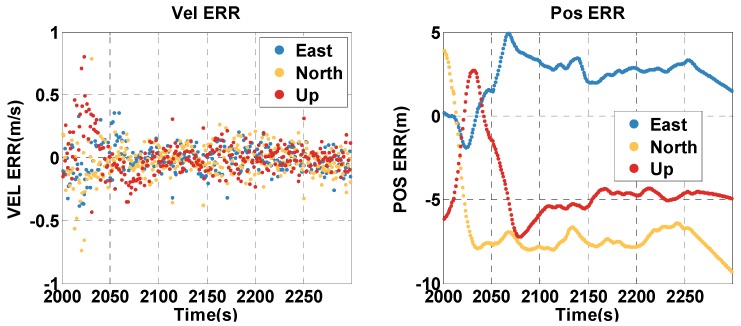
The position and velocity errors of the MTF method in the insufficient satellite case.

**Figure 16:**
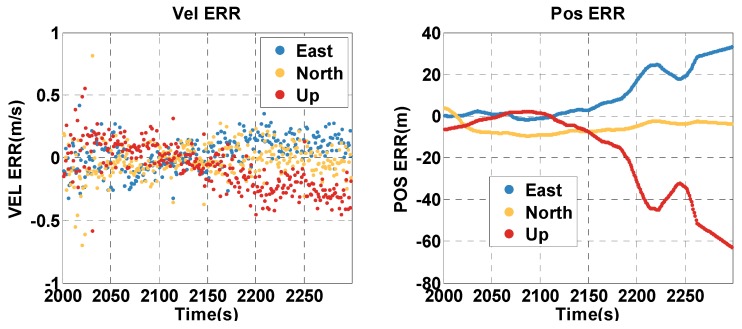
The position and velocity errors of the traditional GNSS/INS tightly coupled integration in the insufficient satellites context in the experimental test.

The vehicle travel in the insufficient satellite context lasts for 6 min (2000–2299 s), and the vehicle moves toward the southeast in Figure 12. As shown in Figure 13, the remaining visible satellites are presented by magenta dots for the epoch of 2000 s. The position and velocity errors of the MTF method in the east, north, and up directions are shown in Figure 15. The errors of the traditional GNSS/INS tightly coupled integration are shown in Figure 16. The errors of the proposed method do not grow over 6 min in Figure 15, but the errors of GNSS/INS tightly coupled integration increase in Figure 16, especially the position error. In the same time, Figure 15 shows that the error of the MTF method is unstable at the beginning of the travel, and the reason is that the new measurements update causes slight fluctuation in the filter. The error statistics are shown in Table 5, and they are characterized by the 95th percentile. Comparing the MTF method with GNSS/INS, the horizontal position errors of the two methods are approximately 8.73 m against 31.55 m, the height errors are approximately 6.66 m against 57.93 m, the horizontal velocity errors are approximately 0.35 m/s against 0.32 m/s, and the vertical velocity errors are approximately 0.32 m/s against 0.40 m/s. Specifically, the statistical horizontal velocity error of the MTF method is a little higher, which is caused by the filter fluctuating at the beginning of the insufficient satellites context when introducing the new measurement update of the barometer geocentric, and the error converges quickly. In sum, the accuracy of the MTF method is close to that in the open-sky context, and performs obviously better than GPS/INS tightly coupled integration.

**Figure 17:**
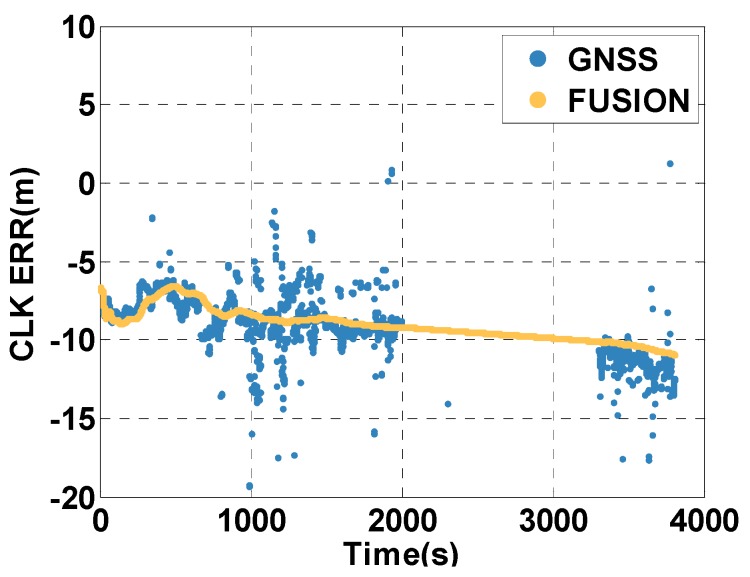
Clock error estimated by the MTF method and by GNSS alone.

In the case of insufficient satellites, the MTF method predicts the clock error by the clock model. Figure 17 shows the comparison between the clock error estimated by the MTF method and that calculated by GNSS resolution. During 2000–3299 s, there is no GNSS result for insufficient satellites. The clock model works well, as the predicted clock error fits the actual clock error well, before 2000 s and after 3299 s.

### 4.3. Satellite Outage Case

**Figure 18:**
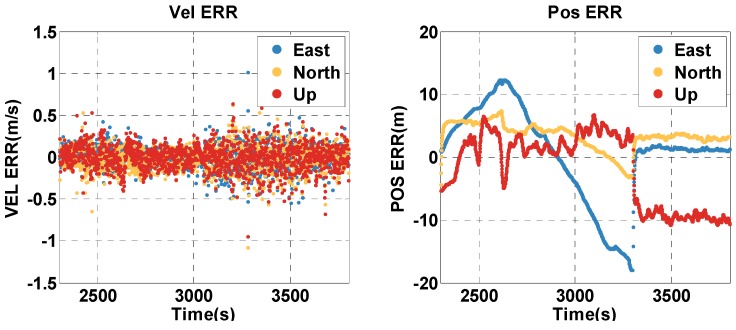
The position and velocity errors of the proposed MTF method in the satellite outage case.

**Figure 19:**
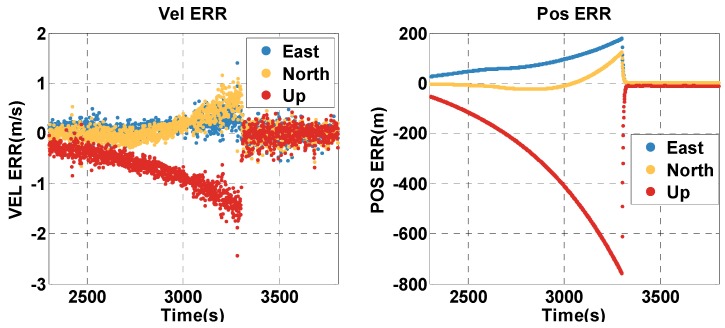
The position and velocity errors of the traditional GNSS/INS tightly coupled integration in the satellite outage case.

The vehicle travels in the satellite outage context for about 16 min (2300–3299 s). The position and velocity errors of the MTF method are shown in Figure 18. The position and velocity errors of the traditional GNSS/INS tightly coupled integration are shown in Figure 19. The errors of both methods increase over time, but that of the MTF method is much slower. In addition, the height error does not grow over time. The error statistics are shown in Table 6. The errors are characterized by the 95th percentile. Comparing the MTF method with GNSS/INS, the horizontal position errors of the two methods are approximately 15.52 m against 184.4 m, the height errors are approximately 5.34 m against 685.8 m, the horizontal velocity errors are approximately 0.34 m/s against 0.78 m/s, and the vertical velocity errors are approximately 0.27 m/s against 1.40 m/s. The MTF method has obvious advantages in both the horizontal and height directions by the aiding of the odometer and barometric altimeter.

## 5. Conclusions

To improve the continuity and reliability of vehicle navigation in complex urban environments, we presented a multi-sensor tight fusion (MTF) method. The fusion method considers GNSS, INS, odometer, barometric altimeter, and clock model sensors, which are easily obtained for vehicles and complement each other. The method designs distinct strategies for the open-sky, insufficient satellites, and satellite outage contexts. In detail, in the open-sky context, the sensor errors can be correctly estimated, which is important as the GNSS signal is impaired. In the insufficient satellites context, the barometric altimeter is treated as a geocentric satellite, combing with the clock model to achieve measurement updates to improve the position accuracy. In the satellite outage context, the odometer and barometric altimeter are used to reduce the horizontal and height divergence of the navigation system. In addition, to reduce the cost of setting up a barometric reference station, the method estimates the error of the barometric altimeter when the GNSS are in good satellite visibility conditions. In sum, the MTF method performs better by adding on other available sensors and a specific fusion strategies design, compared with the traditional GNSS/INS tightly coupled integration.

The simulation test and real-device test were designed to validate the performance of the MTF method. In the simulation test, the sensor errors are preset, and the result shows that the sensor errors estimated by the MTF method match with preset parameters in the open-sky context. In the simulation test and real-device test, the position errors almost do not grow in the insufficient satellites context for 6 min. In the satellite outage context, the position error divergence decreases obviously compared with the traditional GNSS/INS tightly coupled integration. While the validation tests in this paper show that the MTF method performs well, in the future, the method should be modified and optimized to adapt to the actual environment. 

## Figures and Tables

**Table 1:** Initial state parameters of the trajectory.

Type	Initial State of the Trajectory
Lan/Lon/Height	34.246048°, 108.909664°, 380 m
Heading/Roll/Yaw	0°, 0°, 0°
East/North/Up velocity	0 m/s, 0 m/s, 0 m/s

**Table 2:** List of sensor simulation parameters.

Sensor	Sensor Error	Sample Rate
GNSS	Pseudorange: Gaussian noise of 1 m. Pseudorange rate: Gaussian noise of 0.1 m/s. Clock model: the clock drift is 1 × 10^−8^, and Gaussian noise of 1 × 10^−11^ is added to the clock drift. The initial clock error is zero.	1 Hz
Accelerometer	Bias of 100 ug, velocity random walk of 10 $ug/\sqrt{Hz}$	100 Hz
Gyroscope	Bias of 0.05 ${}^{{}^{\circ}}/hr$, angular random walk of 1 × 10^−3^ ${}^{{}^{\circ}}/\sqrt{hr}$	1 Hz
Odometer	Scale factor is 0.9, Gaussian noise of 0.1 m/s	1 Hz
Barometric altimeter	Reference pressure is 1000 hPa when the height is zero, Gaussian noise of 0.1 hPa (equivalent to about 1 m)	1 Hz

**Table 3:** Statistical analysis of the errors of the MTF method and the traditional GNSS/INS tightly coupled integration in the insufficient satellites context in the simulation test.

Method	Horizontal Velocity by 95th Percentile (m/s)	Vertical Velocity by 95th Percentile (m/s)	Horizontal Position by 95th Percentile (m)	Height by 95th Percentile (m)
MTF	0.0056	0.0014	0.3417	0.1851
GNSS/INS	0.0087	0.0015	0.6999	0.5227

**Table 4:** Comparison of statistical results generated by the proposed MTF method and conventional GNS/INS coupled integration in the satellite outage case (the 95th percentile).

Method	Horizontal Velocity by 95th Percentile (m/s)	Vertical Velocity by 95th Percentile (m/s)	Horizontal Position by 95th Percentile (m)	Height by 95th Percentile (m)
MTF	0.0332	0.0017	15.04	0.214
GNSS/INS	0.1337	0.0147	49.76	5.713

**Table 5:** Statistical analysis of the errors of the MTF method and the traditional GNSS/INS tightly coupled integration in the insufficient satellites context in the experimental test.

Method	Horizontal Velocity by 95th Percentile (m/s)	Vertical Velocity by 95th Percentile (m/s)	Horizontal Position by 95th Percentile (m)	Height by 95th Percentile (m)
MTF	0.35	0.32	8.73	6.66
GNSS/INS	0.32	0.40	31.55	57.93

**Table 6:** Statistical analysis of the errors of the MTF method and the traditional GNSS/INS tightly coupled integration in the satellite outage context in the experimental test.

Method	Horizontal Velocity by 95th Percentile (m/s)	Vertical Velocity by 95th Percentile (m/s)	Horizontal Position by 95th Percentile (m)	Height by 95th Percentile (m)
MTF	0.34	0.27	15.52	5.34
GNSS/INS	0.78	1.40	184.4	685.8

## Appendix A

(A1) $$M_{aa}=-\left(\omega_{ie}^{n}+\omega_{en}^{n}\right)\times$$

(A2) $$M_{av}=\left[\begin{array}{ccc} 0 & -1/\left(Rm+h\right) & 0\\ 1/\left(Rn+h\right) & 0 & 0\\ 1/\left(Rn+h\right)\cdot \tan(Lan) & 0 & 0 \end{array}\right]$$

(A3) $$M_{ap}=\left[\begin{array}{ccc} 0 & 0 & v(2)/{\left(Rm+h\right)}^{2}\\ -\omega_{ie}^{n}\left(3\right) & 0 & -v(1)/{\left(Rn+h\right)}^{2}\\ \omega_{ie}^{n}\left(2\right)+v(1)/(Rn+h)\cdot \sec^{2}(Lan) & 0 & -v(1)/{\left(Rn+h\right)}^{2}*\tan(Lan) \end{array}\right]$$

(A4) $$M_{va}=\left(C_{b}^{n}\cdot f_{b}\right)\times$$

(A5) $$M_{vv}=\left(v\right)\times M_{av}-\left(2\omega_{ie}^{n}+\omega_{en}^{n}\right)\times$$

(A6) $$M_{vp}=v\times \left[\begin{array}{ccc} 0 & 0 & v(2)/{\left(Rm+h\right)}^{2}\\ -2\omega_{ie}^{n}(3) & 0 & -v(1)/{\left(Rn+h\right)}^{2}\\ 2\omega_{ie}^{n}(2)+v(1)/(Rn+h)\cdot \sec^{2}(Lan) & 0 & -v(1)/{\left(Rn+h\right)}^{2}\cdot \tan(Lan) \end{array}\right]$$

(A7) $$M_{pv}=\left[\begin{array}{ccc} 0 & 1/\left(Rm+h\right) & 0\\ 1/\left(Rn+h\right) & 0 & 0\\ 0 & 0 & 1 \end{array}\right]$$

(A8) $$M_{vp}=\left[\begin{array}{ccc} 0 & 0 & -v(2)/{\left(Rm+h\right)}^{2}\\ v(1)/(Rn+h)\cdot \tan\left(Lan\right)\sec\left(Lan\right) & 0 & -v(1)/{\left(Rn+h\right)}^{2}\cdot \sec\left(Lan\right)\\ 0 & 0 & 0 \end{array}\right]$$

(A9) $$\omega_{ie}^{n}=\left[\begin{array}{c} 0\\ \omega_{ie}\cos\left(Lan\right)\\ \omega_{ie}\sin\left(Lan\right) \end{array}\right]$$

(A10) $$\omega_{en}^{n}=\left[\begin{array}{c} -\frac{v\left(2\right)}{Rm+h}\\ \frac{v\left(1\right)}{Rn+h}\\ \frac{v\left(1\right)}{Rn+h}\tan\left(Lan\right) \end{array}\right]$$

where Rm is the radius of curvature along the meridian circle, and Rn is the radius of curvature along the meridional circle. Lan is the longitude, h is the height, and *v* is the velocity in the local frame. $\omega_{ie}$ is the earth’s rotation angular velocity, and $f_{b}$ is the specific force from the accelerometer sensor.
